# *RNF213* vasculopathy manifested in various forms within a family: A case report

**DOI:** 10.1097/MD.0000000000036627

**Published:** 2023-12-15

**Authors:** Seong-Soo Lim, Sangshin Park, Byeong Ho Oh, Kiwook Jung, Jang-Whan Bae, Dae-Hwan Bae

**Affiliations:** a Department of Internal Medicine, Chungbuk National University Hospital, Cheongju, South Korea; b Division of Cardiology, Department of Internal Medicine, Chungbuk National University Hospital, Cheongju, South Korea; c Department of Neurosurgery, Chungbuk National University Hospital, Cheongju, South Korea; d Department of Laboratory Medicine, Chungbuk National University Hospital, Cheongju, South Korea.

**Keywords:** genetics, Moyamoya disease, *RNF213*, vasculopathy

## Abstract

**Rationale:**

The ring finger protein 213 (*RNF213*) p.R4810K variant has been identified as being associated with Moyamoya disease (MMD), a condition that is more prevalent in East Asians. This association extends beyond cerebral vessels and has been implicated in coronary artery disease.

**Patient concerns:**

A 36-year-old female was admitted to the emergency room with chest pain. Although the patient had no known underlying conditions or risk factors for atherosclerosis, she was diagnosed with unstable angina and underwent percutaneous coronary intervention. Given her older sister’s ongoing treatment for MMD, it was suspected that the patient’s coronary artery disease might be linked to the MMD-associated gene mutation.

**Diagnoses:**

Coronary angiography revealed 80% narrowing of the proximal left anterior descending artery. Based on clinical symptoms and coronary angiography, we diagnosed it as unstable angina.

**Intervention:**

Due to the family history of MMD and detection of the *RNF213* p.R4810K heterozygous variant in the patient’s older sister, genetic counseling was recommended. Next-generation sequencing for vascular diseases was performed.

**Outcomes:**

Genetic testing confirmed the presence of an *RNF213* p.R4810K heterozygous variant in the patient, mirroring that in her sister. An *RNF213* p.C4397R heterozygous variant was identified concomitantly, although it was categorized as a variant of uncertain significance. Coronary artery disease has been attributed to the *RNF213* p.R4810K variant.

**Lessons:**

Although MMD is rare in Western populations, it is more common in East Asian populations. Traditionally, MMD diagnoses have focused solely on the cerebral vessels without guidelines for the assessment of other vascular involvements. This familial case underscores the fact that a single genetic mutation can manifest in diverse ways in different diseases. Hence, the need and regularity of systemic vessel screening should be thoughtfully considered in such a context.

## 1. Introduction

Moyamoya disease (MMD) is characterized by unexplained narrowing or occlusion of the terminal portion of the internal carotid arteries, specifically at the origins of the anterior and middle cerebral arteries. Adjacent to these narrowed areas, small aberrant blood vessels, known as moyamoya vessels can be observed.^[1]^

A significant correlation was observed between MMD and the p.R4810K variant in the ring finger protein 213 (*RNF213*) gene located on chromosome 17q25.3. The *RNF213* p.R4810K variant is a frequent genetic variant in the East Asia, with particularly high prevalence in Korea and Japan.^[2,3]^ Notably, approximately 80% of the patients with MMD in Korea and 90% of the patients in Japan harbor this mutation. However, 2.7% of Korean and 2.5% of Japanese individuals without MMD also exhibit this variant.^[4]^ These statistics indicate a potentially low penetrance rate.

The prevalence of MMD is considerably lower in regions outside of East Asia. Moreover, cases with the *RNF213* p.R4810K variant that display autosomal dominant inheritance patterns often manifest MMD at a younger age when homozygous. Such patients may also exhibit more severe conditions, including peripheral pulmonary artery stenosis.^[5]^ There is growing evidence to suggest that this genetic mutation does not solely affect cerebral vessels, but also frequently impacts systemic vessels.

Interestingly, when the variant is heterozygous, its effects can be observed beyond MMD, influencing the extracranial vessels.^[6]^ Among these, numerous reports have highlighted an association between the onset of coronary heart disease and MMD.^[7–10]^ However, there are no reports linking the *RNF213* p.R4810K variant directly to patients with coronary heart disease. Therefore, we aimed to present a case detailing vasculopathy manifesting in diverse forms within a family carrying the *RNF213* p.R4810K variant.

## 2. Case description

**Figure 1.**
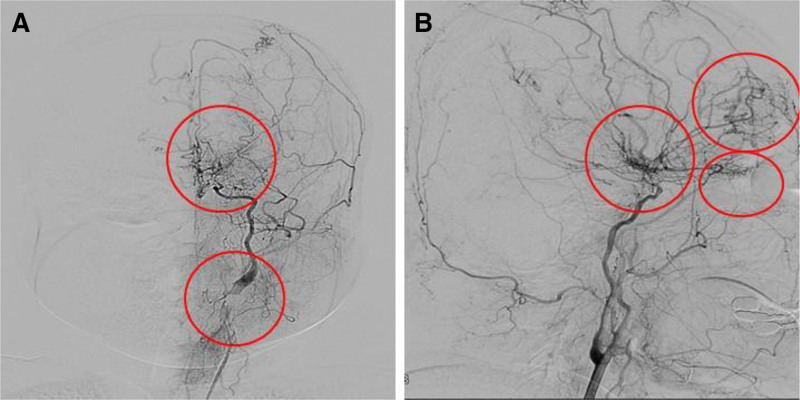
Transfemoral cerebral angiography. (A) The left distal internal carotid artery occlusion and the absence of the middle cerebral artery and anterior cerebral artery. (B) Collateral circulation between the left internal carotid artery and external carotid artery (moyamoya vessels) was observed.

A 36-year-old woman presented to the cardiology department with complaints of chest pain during exercise that had begun 1 week prior to presentation. Although she was a nonsmoker with no known underlying conditions for atherosclerosis, her older sister had been diagnosed with MMD 1 year prior. The diagnosis of the patient’s sister was made when she was admitted to the neurology department for intermittent unilateral motor weakness and aphasia. Transfemoral cerebral angiography of the patient’s older sister revealed occlusion of the left distal internal carotid artery and the absence of both the middle and anterior cerebral arteries. Collateral vessels, known as moyamoya vessels, were also evident between the internal and external carotid arteries (Fig. 1). Targeted gene panel testing for hereditary stroke disease based on next-generation sequencing (NGS) of the patient’s older sister revealed a heterozygous missense variant, NM 001256071.3 (*RNF213*):c.14429G > A (p.R4810K). Consequently, the patient underwent magnetic resonance imaging and magnetic resonance angiography to assess for MMD.

Blood test results revealed no other abnormalities. Cardiac enzymes were within normal ranges: creatine kinase at 70 U/L (reference range 29–145 U/L), CK-MB at 0.942 ng/mL (reference range 0–4.94 ng/mL), and high-sensitivity troponin T at 9 ng/L (reference range < 14 ng/L). Electrocardiography findings were normal, and the echocardiogram showed no regional wall motion abnormalities.

**Figure 2.**
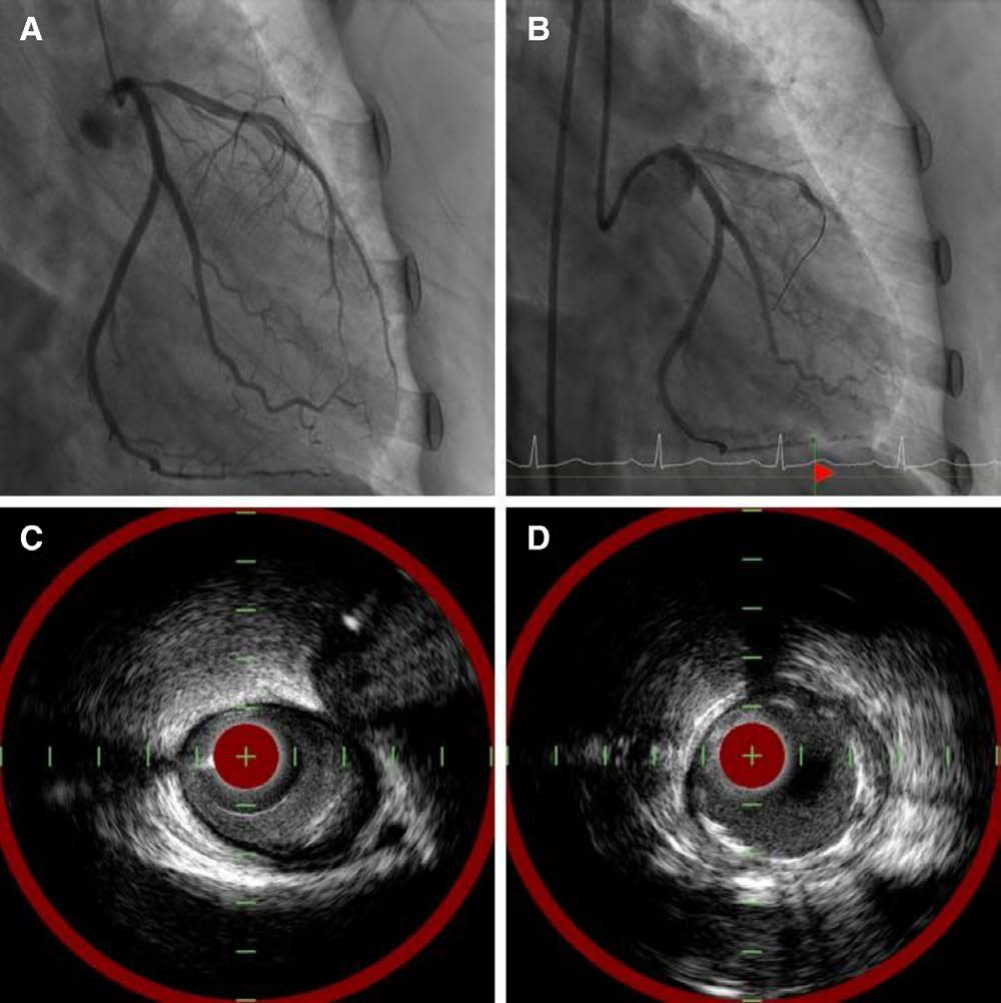
Revascularization of the left anterior descending artery (LAD). (A) Proximal LAD 80% stenosis was seen. (B) Percutaneous coronary intervention at proximal LAD. (C) Prior to the procedure, the intravascular ultrasound (IVUS) showed a minimal lumen area of 2.8 mm^2^. (D) After the procedure, the intravascular ultrasound (IVUS) showed a minima stent area of 6.9 mm^2^, indicating successful optimization.

Despite the patient’s young age and absence of other familial cardiovascular risk factors, the distinctive nature of her chest pain and family history of MMD raised the suspicion of unstable angina. Consequently, coronary angiography revealed 80% narrowing of the proximal left anterior descending artery. Intravenous ultrasonography revealed a minimal lumen area of 2.6 mm^2^, which corresponded to 70% stenosis. Consequently, percutaneous coronary intervention was performed (Fig. 2).

Given her presentation of unstable angina, young age, absence of risk factors, and familial history of MMD, genetic testing was performed. Similar to her older sister, she harbored the *RNF213* p.R4810K variant in heterozygous status. Another heterozygous missense variant, NM 001256071.3(*RNF213*):c.13189T > C (p.C4397R) was also detected. However, this variant was categorized as a variant of uncertain significance and was not considered pivotal for the patient. The brain magnetic resonance imaging and magnetic resonance angiography conducted earlier did not reveal any signs of MMD. After the coronary intervention, the patient was discharged without complications. During the subsequent outpatient visits, the patient remained on medication and reported no episodes of angina or chest pain.

## 3. Discussion

In 2011, it was discovered that *RNF213* mutations play a pivotal role in the genetic basis of MMD.^[11]^ The *RNF213* gene is responsible for encoding a substantial protein comprising 5207 amino acids, weighing 584 kDa.^[12]^ Its intricate structural characteristics contribute to the scarcity of biochemical data, making it challenging to pinpoint the exact mechanism that triggers MMD. However, electron microscopic observations in rat experiments revealed a distinct protein structure: one end extending into a long arm, the center harboring an ATPase motor within a looped section, and the other end housing an E3 enzyme module.^[13]^ These insights indicate that mutations within the *RNF213* gene result in a diminished E3 enzyme and motor protein, subsequently influencing hypoxia, fat metabolism, NF-kB signaling, and angiogenesis.^[14]^ Consequently, the *RNF213* gene mutation does not affect only cerebral vessels; it may influence systemic vessels, predisposing individuals to diverse vascular ailments. A homozygous mutation, compared to its heterozygous counterpart, results in a more aggressive and precocious vascular disease, likely due to a pronounced reduction in E3 enzyme and motor.^[15,16]^

The primary vessel affected by this mutation is the aorta. In individuals with the heterozygous *RNF213* p.R4810K variant, no notable abnormalities were observed in the aorta. Conversely, those homozygous for this variant exhibited diffuse aortic constrictions. Previously published literature suggested that such vascular manifestations may predispose patients to inherited aortic diseases, including Williams syndrome and coarctation of the aorta.^[16,17]^

Additionally, the *RNF213* p.R4810K variant is associated with peripheral pulmonary artery stenosis.^[5]^ While such stenosis is also observed in conditions such as chronic thromboembolic pulmonary hypertension and pulmonary tumor thrombotic microangiopathy, the constriction in these instances stems from inflammatory reactions spurred by blood clots and malignant cells.^[18,19]^ Differentiating these conditions using computed tomography, lung ventilator/perfusion scans, and other techniques is complicated. Therefore, diagnostics often rely on genetic testing and, where necessary, pulmonary angiography. Peripheral pulmonary artery angioplasty may be used as a therapeutic measure in cases of extreme PH.^[20]^

Notably, 4.6% of patients with MMD have symptomatic coronary heart disease.^[10]^ What sets these patients apart is the early onset of symptoms and the lack of other cardiovascular risk factors, such as hypertension, hyperlipidemia, and diabetes. Hence, a plausible correlation with MMD is proposed. Particularly, in homozygous mutations, the disease surfaces at a tender age, typically with coronary ostial disease and spastic coronary arteries.

In 1 documented case, a young woman without cardiovascular risk factors developed coronary artery disease. Although she and her older siblings possessed both *RNF213* p.R4810K heterozygous variants, only the latter exhibited MMD symptoms. Brain imaging revealed no indication of MMD. While prior research has acknowledged the concurrence of significant coronary artery disease in MMD, no current insights exist on the evaluation of coronary artery disease in individuals with the *RNF213* p.R4810K variant but without MMD. Recent guidelines have focused on genetic counseling for conditions such as familial hypercholesterolemia, hypertrophic cardiomyopathy, and dilated cardiomyopathy.^[21–23]^ Depending on these factors, various therapeutic strategies are being pursued in ongoing gene therapy research. However, guidelines specifically addressing genetic counseling for coronary are lacking. Given the prominent occurrence of the *RNF213* p.R4810K variant in East Asians and its unanticipated high penetrance, the presence of a familial history of *RNF213* vasculopathy may necessitate preventive strategies against complications and fatalities from various vascular diseases via genetic counseling and vasculopathy surveillance.

## 4. Conclusion

Our study highlights the unique presentation of the *RNF213* p.R4810K variant, revealing manifestations of diverse forms of vasculopathy within a single-family lineage. Although the diagnostic and therapeutic approach utilized for our primary patient mirrored the standard protocols for MMD and coronary artery disease, our findings underscore the intricate relationship between genetics and vascular health. Specifically, this study highlights the potential for various vascular pathologies to emerge within a single-family tree. Moreover, when young individuals present with coronary artery disease in the absence of conventional cardiovascular risk determinants, clinicians may need to delve deeper into the potential genetic underpinnings. As we deepen our understanding of how the *RNF213* gene mutation precipitates vasculopathy, we foresee a surge in research efforts aimed at predicting the presentation of vasculopathy and exploring gene therapy interventions.

## Acknowledgments

We appreciate the hospital staff for their support and assistance and thank them for their understanding. We also thank the participants of the study for their cooperation over the course of the study.

## Author contributions

**Conceptualization:** Jang-Whan Bae, Dae-Hwan Bae.

**Data curation:** Byeong Ho Oh, Kiwook Jung, Jang-Whan Bae.

**Formal analysis:** Sangshin Park, Jang-Whan Bae, Dae-Hwan Bae.

**Investigation:** Byeong Ho Oh, Kiwook Jung.

**Project administration:** Dae-Hwan Bae.

**Resources:** Jang-Whan Bae.

**Writing – original draft:** Seong-Soo Lim, Sangshin Park.

**Writing – review & editing:** Dae-Hwan Bae.
